# Fast screening method to identify salinity tolerant strains of foliose *Ulva* species. Low salinity leads to increased organic matter of the biomass

**DOI:** 10.1007/s10811-024-03222-0

**Published:** 2024-04-03

**Authors:** Clara Simon, Antoine Fort, Diane Jouanneau, Marcus McHale, Ronan Sulpice

**Affiliations:** 1 School, Plant Systems Biology Lab, Ryan Institute & Marei Centre for Marine, Climate and Energy, School of Biological & Chemical Sciences, University of Galway, Galway, Ireland; 2 Department of Veterinary and Microbial Sciences, Technological University of The Shannon: Midlands, Athlone, Ireland; 3 Sorbonne Université, CNRS, Laboratory of Integrative Biology of Marine Models (LBI2M), Station Biologique de Roscoff, Roscoff, France

**Keywords:** *Ulva* spp., Strain selection, Low salinity, Growth, Nutritional value

## Abstract

Sea lettuce (*Ulva*) is recognised for its potential in food, pharmaceutical, nutraceutical, biorefinery and bioremediation industries and is increasingly being cultivated. The requirements of those industries vary widely in terms of biomass composition. *Ulva* biomass composition and growth is known to be directly influenced by environmental factors, e.g., temperature, light, salinity, nutrient availability as well as by genetic factors and likely by microbiome composition. In order to select for the highest yielding strains in a given environment, we tested the suitability of common-garden experiments, i.e., the co-cultivation of different strains grown under shared conditions. Fifteen strains from six different foliose *Ulva* species were grown together under two different salinities, 35 ppt and 15 ppt. After 32 days, only *U. australis* strains remained at both salinities. If selection at low salinity was mostly based on survival, the selection process at seawater salinity was driven by competition, largely based on growth performance. Growth rates after a month were very similar at both salinities, suggesting the *U. australis* strains cope equally well in either condition. However, the composition of the biomass produced in both environments varied, with the content of all organic compounds being higher at low salinity, and the ash content being reduced in average by 66%. To summarize, this study provides an established bulk-selection protocol for efficiently screening large numbers of locally-sourced strains and highlights the potential of low salinity treatments for increased organic matter content, particularly in carbohydrates.

**Supplementary Information:**

The online version contains supplementary material available at 10.1007/s10811-024-03222-0.

## Introduction

*Ulva* has long been recognised for its valuable biomass and in recent years its use has become increasingly popular with a wider range of applications. *Ulva* biomass can be used in the food and feed sectors and contains valuable metabolites, including bioactive compounds, that have interest for the pharmaceuticals, nutraceuticals or biorefineries industries (Mabeau and Fleurence 1993; Barbier et al. 2019). In addition, *Ulva* spp. can be used for bioremediation, for example as part of integrated multi-trophic aquaculture (Naldi and Wheeler 2002; Cocquyt et al. 2010; Yokoyama and Ishihi 2010; Nielsen et al. 2012).

The selection of *Ulva* strains should be performed carefully, taking into account their ecophysiological characteristics, as well as the biomass use and market value (Neori et al. 2004). Chemical composition, growth rate and/or nutrient removal efficiency are essential traits when it comes to selecting the right strain (Santos 2006). In an aquaculture context, a fast-growing strain will often be favoured, but its nutritional and sensory value will also be of importance for the market value of the produced biomass.

*Ulva* spp. are often the inhabitants of biotopes characterised by changing salinities and their study might help to learn more about the mechanisms allowing for acclimatisation and tolerance to salt. Salinity is one of the main environmental factors influencing species diversity and richness of macroalgae living in coastal areas (Rybak 2018). The genus *Ulva*, growing mainly in the intertidal zone, is known to be very tolerant to variations in salinity levels, from hypohaline to hyperhaline (Zaneveld 1969; Lartigue et al. 2003; Xiao et al. 2016; Rybak 2018). However, not all *Ulva* species are equal in their ability to tolerate a wide range of salinities. It has been suggested, both in laboratory and in ecological surveys, that the morphotype, i.e. tubular or foliose thallus, plays an important role in adaptation and tolerance to salinity variations. Rybak (2018) described that tubular *Ulva* spp. can be found in both fresh and saline waters, whereas foliose *Ulva* are only found in saline waters. The ability of foliose *Ulva* spp. to adapt and survive hyposaline conditions is more limited in contrast to tubular *Ulva* spp. which seem to tolerate a wider range of salinity variation (McAvoy and Klug 2005; Gao et al. 2016; Xiao et al. 2016). Interestingly, some species can show different morphologies in different locations. *U.* *compressa* and *U. intestinalis* can be found with two distinct morphotypes, tubular and foliose thalli (Blomster et al. 2002). *Ulva compressa* is found as a monostromatic tubular morphotype in a saline/hypersaline environment and a distromatic foliose form in a low salinity environment, such as estuarine sites (Hofmann et al. 2010; van der Loos et al. 2022). Thus, this observation would suggest that the morphotype by itself is not responsible for adaptation to low salinity environments, but that tubular *Ulva* species possess specific attributes, independent of those that shape their morphotype, that allow them to be more tolerant of low salinity environments.

Shifts from low to high salinities and vice versa for periods of one to six days have been shown to cause a significant reduction in growth and oxidative cell damage in *Ulva* (Lu et al. 2006; Luo and Liu 2011). Variations in osmolarity and ion concentrations in the media disturb turgor pressure and ion distribution and thus the metabolism in the cell, and can lead to an accumulation of reactive oxygen species which cause damages to macromolecules such as proteins and disturb growth (Kirst 1990; Karsten et al. 1991; Wu and Lee 2008; Liu and Pang 2010). However, the impact of long-term hypo/hyper salinity (i.e., continuous exposure over a period of weeks) on metabolism and growth of *Ulva* spp. has been rarely investigated (Lartigue et al. 2003; Choi et al. 2010).

The present study demonstrates the use of batch culture for strain selection based on survival and competition, probably through exploitative competition for common limited resource (indirect interaction) and likely unknown mechanism of interference competition (direct interaction) (Holomuzki et al. 2010). We performed common-garden experiments (Berend et al. 2019), by growing together 15 strains from 6 different foliose *Ulva* species from different geographic locations under two different salinities, seawater salinity (35 ppt) and brackish water salinity (15 ppt) with the hypothesis that the fastest-growing strain should dominate in mixed cultures. After 32 days, when both cultures had achieved a density of >3g L^-1^, excess biomass was removed weekly and analysed for its biochemical content. The presence of the species was monitored over a period of 70 days. In parallel, all strains used for the common-garden experiments were also tested independently for their growth, in order to assess if the selection process occurring could be explained by variations in growth performances.

## Materials and methods

### Seaweed material

Intertidal foliose *Ulva* samples were collected at various sampling sites in Galway Bay (Ireland) and Brittany (France) (Supplementary Table S1). Immediately after collection, samples were placed in bags filled with seawater and brought back to the lab in a coolbox. Samples collected outside Ireland were shipped in individual Falcon tubes filled with seawater inside coolboxes. Upon arrival in the laboratory, the tissue samples were wiped with paper tissue to remove possible epiphytes and rinsed with artificial seawater. The holdfast was removed to ensure better homogeneity of the tissue, and the remaining thalli were placed in 500 mL glass bottles filled with media containing artificial seawater (37 g L^-1^; Red Sea Coral Pro) and 1X Cell-HI F2P nutrients (Varicon Aqua; [N]=[P]=1mM), at a constant temperature of 15±1°C (this temperature was chosen according the natural environment temperature) under fluorescent light (Osram T5 tubes) at 200 μmol photons m^-2^s^-1^ photosynthetically active radiation and a light:dark photoperiod of 12h:12h. All *Ulva* samples were maintained in vegetative growth for at least three weeks at 35 ppt prior the start of the experiments to ensure the acclimation of all strains to the growth conditions used in this study.

### Experiment design

#### Common-garden experiment

This competition experiment consisted of a batch cultivation of 15 foliose *Ulva* strains in one 5 L conical flask at 35 ppt, the salinity of where the strains were collected and maintained in the laboratory and another 5-L conical flask at 15 ppt, representing the low salinity environment. For this experiment we used 6 *Ulva* *lacinulata*, 4 *Ulva* *uncialis* (previously named *Ulva rigida*; Bachoo et al. 2023), 2 *Ulva* *australis*, 1 *Ulva* *compressa*, 1 *Ulva* *fenestrata* and 1 *Ulva rigida* (details of the strains in Supplementary Table S1). The medium was same as described above, (Red Sea Coral Pro salts) and added at different concentrations to obtain the required salinity levels (37 g L^-1^ for 35 ppt; 16 g L^-1^ for 15 ppt). The two flasks were placed in a constant temperature room (15°C ± 1°C), under a light intensity of 200 μmol photons m^-2^s^-1^ photosynthetically active radiation with Spectron T8 LED 1.5 GB tubes (HydroGarden), a photoperiod of 12h:12h and aeration. Two 113 mm^2^ discs per strain were used for the start of the experiment, which correspond to around 50 mg biomass L^-1^ in total. The media were changed two times per week to ensure that nutrients were always in excess. The fresh weight of the *Ulva* biomass was determined once a week at the end of the day, after removal of excess water by blotting dry on tissue paper. When the weighed biomass reached more than 15 g (corresponding to of a density of 3 g biomass L^-1^) only 10 g were returned to cultivation and the surplus biomass flash frozen in liquid nitrogen and stored at -80°C prior to biochemical and genetic analyses. For this reason, no biomass was removed before day 32 as the density was below the established density threshold of 3 gFW L^-1^. After day 32, the weekly weighed biomass in excess of 10 g was stored for genetic and biochemical analyses. The experiments were conducted over a total of 70 days.

#### Individual determination of growth rates

The 15 foliose *Ulva* strains, 1 *U.* *fenestrata*, 6 *U. lacinulata*, 4 *U.* *uncialis*, 2 *U. australis*, 1 *U. rigida* and 1 *U.* *compressa* were gown as well under the phenotyping platform described by Fort et al., (2019) at a salinity of 35 ppt and 15 ppt for one week to assess their growth rates.

### DNA extraction and genetic analysis

DNA extraction was performed using magnetic beads following the method described by Fort et al., (2018). DNA extraction was done in duplicate for each time point. The species identification from the bulk biomass over the time was realised by using the Cleaved Amplified Polymorphic Sequences (CAPS) assay described by Fort et al., (2021). This assay supports characterisation of species composition in an *Ulva* biomass bulk without the need for barcoding. These CAPS assays use restriction enzyme digestion of PCR products to detect the presence (or absence) of specific SNPs in the ITS1 barcode. This method allows us to realise a qualitative determination of *Ulva* species present in bulk biomass. For this study, 4 different enzymatic digestions with different enzymes (all from Fisher Scientific) were conducted. For all the digestion reactions Buffer 2 (NEB) and the endonuclease T7 (New England Biolabs) were used. The first enzymatic digestion was done with the enzyme specific to *U.* *australis*, BtscI. Then, CvQI/BtscI, enzyme specific to *U.* *australis* and *U.* *fenestrata*. The next one was carried out with BfaI+BamHI, the enzyme specific to *Ulva* *spA* and *U.* *lacinulata*. Finally, the PspoMI enzyme specific to *U.* *compressa *was used.

### Growth monitoring and metabolic analysis

*Ulva* growth in the garden experiments was monitored by weekly fresh weight measurements and expressed as the Specific Growth Rate (SGR, in % day^-1^) calculated using the following formula:

$$SGR[\mathrm{\%}\ {{\text{day}}}^{-1}]=\frac{{\text{ln}}\left({Fresh\; Weight}_{t}\right)-{\text{ln}}\left({Fresh\; Weight}_{\left(t-1\right)}\right)}{t-\left(t-1\right)}\times 100$$

Biochemical analyses from samples collected each week after 32 days of cultivation were conducted. Freeze-dried biomass samples were ground into powder using a ball mill (Qiagen TissueLyser II) and analyses were performed on ~4 mg aliquots. Soluble metabolites were extracted from aliquots by sequential incubation for 30 min at 90 °C in 100% ethanol, 80% ethanol and 50% ethanol (Esteves-Ferreira et al. 2017). The supernatants obtained were used for pigment and amino acids content and the pellet was used to quantify the protein, starch and ulvan content.

Protein was determined according to Lowry et al. (1951) and amino acids according to Yemm et al. (1955). Starch was determined according to Smith and Zeeman (2006) by measuring the amount of glucose released after enzymatic starch degradation. Ulvan content was determined by digestion overnight at 37°C of the ulvan content by an ulvan lyase enzyme in HEPES buffer (0.1 M; pH 7.5) and subsequent spectrophotometric determination at 240 nm. All assays were performed using 96-well plates. To control for potential inter-plate variations during metabolite assays, two aliquots from a large pool of *Ulva* biomass were added to all the assay plates. Ash content in *Ulva* tissue samples was measured by combusting ground freeze-dried disc samples at 550°C for 12 h.

### Data analysis

All data were analysed using R (R Core Team 2020). Biochemical data are presented as mean ± standard deviation (SD) of triplicate extraction. Specific growth rate data are presented as mean ± SD of three biological replicates.

Statistical differences for time series on SGR for the common-garden experiment between low and seawater treatment were determined using a fixed-effects ANOVA with a confidence level of 95%.

Differences between means were evaluated for significance at *p*-value < 0.05 by using Tukey’s post-hoc test.

Statistical differences on biochemical compounds between low and seawater salinity biomasses for each timepoint were determined using a two-way ANOVA. Differences between means were evaluated for significance at *p*-value < 0.05 by using Tukey’s post-hoc test (TukeyHSD).

Statistical differences among SGR strains were tested using the one-way ANOVA test. A Tukey post-hoc test was then performed after the ANOVA (TukeyHSD).

The conditions of application for the two-way and one-way ANOVA tests were checked using the Shapiro test (normal distribution) and the F test or Bartlett’s test (homogeneity of variance).

## Results

### Growth rates and selection process

#### Common-garden experiment

**Fig. 1:**
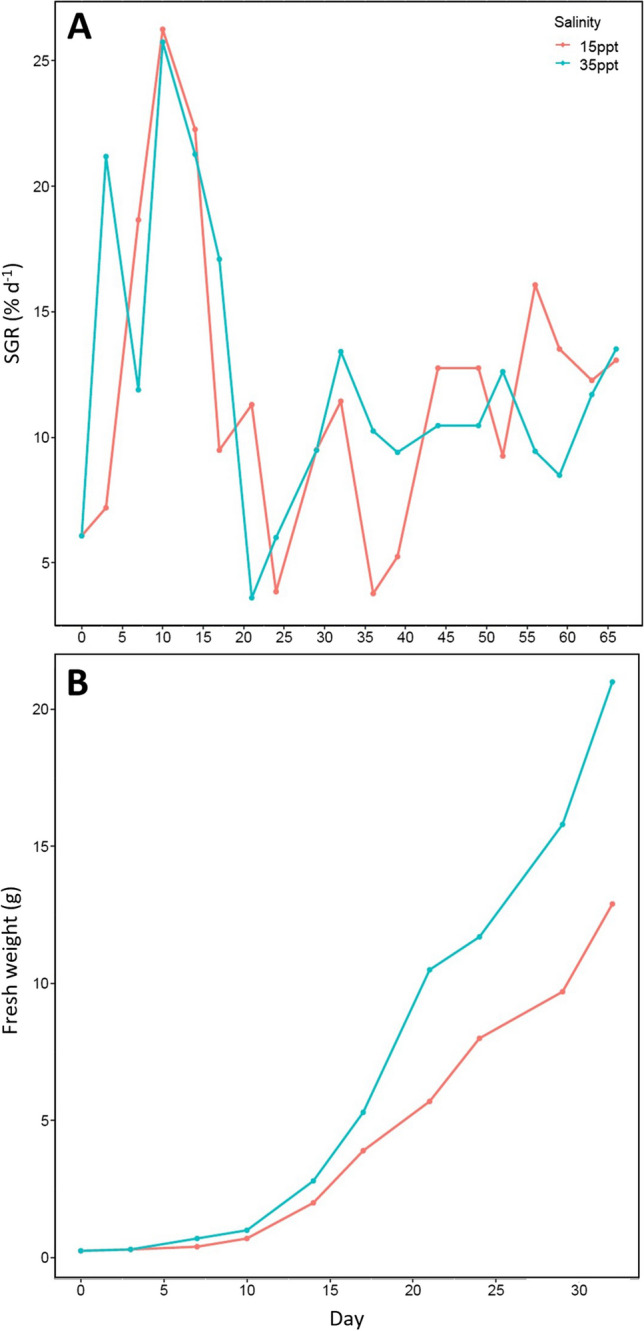
(**A**) Evolution of the specific growth rate (% day^-1^) during the batch culture experiment at 15 ppt and 35 ppt. (**B**) Fresh weight of the two *Ulva* batch cultures at 15 ppt and 35 ppt during the first 33 days of cultivation before any biomass was removed; No significative differences observed between the two salinities overtime (Fixed-effects ANOVA, *p*>0.05)

The specific growth rate of *Ulva* biomass fluctuated during the first month of cultivation at both salinities (Fig. 1). In the period prior to biomass removal (day 0 to 32, Fig. 1B) growth rates were similar across salinities, with a mean growth rate of the low salinity biomass of 12.7 % day^-1^, and 13.6 % day^-1^ for the biomass at 35 ppt (Fig. 1A). After this fluctuating period of growth, from around the 30^th^ day of cultivation, the growth rate started to stabilise at around 11% day^-1^ for both salinities (10.9% day^-1^ for the biomass at 15 ppt and 10.8% day^-1^ for the biomass at 35 ppt, the difference not being statistically different (Fixed-effects ANOVA, p>0.05)).

#### Individual culture experiment

##### Growth rate under seawater condition (35ppt)

The growth rate was assessed in the 35 ppt condition for each strain grown in individual cultures for a month and then transferred on a phenotyping platform at 35 ppt for a week (Fort et al. 2019). The growth rates obtained are presented in the Table 1. *Ulva australis* was the species showing the highest growth rate, around 15% day^-1^. The *U.* *lacinulata* strains did show the lowest growth rates and 3 strains among the 6 tested sporulated and died prior being transferred to the phenotyping platform.

**Table 1:** Mean specific growth rate (% day^-1^) of 15 strains from 6 different *Ulva* species when cultivated individually at 35 ppt for 1 month (35 ppt) and then transferred to a phenotyping platform (Fort et al. 2019). Data represent the SGR measured on the fourth week of cultivation ± standard deviation. The values with different superscript letters in a column are significantly different (ANOVA, *p* <0.05)

Strain	Species	SGR (% day^-1^)
CORA7	*U. lacinulata*	sporulated
CORA5	*U. lacinulata*	sporulated
CORA9	*U. lacinulata*	sporulated
CAL13	*U. lacinulata*	2.1±0.4^a^
CO8	*U. lacinulata*	2.1±2.2^a^
CORA11	*U. lacinulata*	11.9±4.4^ab^
CORR8	*U. uncialis*	8.7±6.8^ab^
CORR10	*U. uncialis*	8.6±1.4^ab^
CO7	*U. uncialis*	5.9±0.2^ab^
CAL10	*U. uncialis*	6.8±3.3^ac^
BLD17	*U. australis*	15.3±5.0^b^
HALAUS2	*U. australis*	15.5±1.0^ab^
SIL32	*U. compressa*	14.7±1.6^bc^
TRAM84	*U. fenestrata*	9.8±3.1^ab^
CAL7	*U. rigida*	7.2±3.0^ab^

##### Growth rate under brackish water condition (15 ppt)

The growth rate was assessed in the 15 ppt condition for each strain grown in individual cultures for a month and then transferred on a phenotyping platform at 15 ppt for a week to asses their growth rates. The growth rates obtained are presented in the Table 2. After 2 weeks of experimentation, 4 *U. lacinulata* specimens and all of the *U.* *uncialis* died after sporulating, with no new spore growth being observed, probably because the spores/gametes were not given the opportunity to attach due to the lack of substrate and rapid water movement due to aeration. Then, after three weeks of culture at low salinity, the *U. fenestrata* strain and the last strain of *U. lacinulata* also died. The last individuals growing after 1 month were the 2 *U. australis* strains and the *U. compressa* strain, which were then transferred to the phenotyping platform. The growth rates of those strains were not significantly different (ANOVA, p<0.05; Table 2).

**Table 2:** Mean specific growth rate (% day^-1^) of the *U. compressa* strain and the two *U. australis* strains when cultivated individually at brackish salinity (15 ppt) for one month and then transferred onto a phenotyping platform. The values with different superscript letters in a line are significantly different (ANOVA, *p* <0.05)

Strain	Species	SGR (% d^-1^)
CORA7	*U. lacinulata*	sporulated
CORA5	*U. lacinulata*	sporulated
CORA9	*U. lacinulata*	sporulated
CAL13	*U. lacinulata*	sporulated
CO8	*U. lacinulata*	sporulated
CORA11	*U. lacinulata*	sporulated
CORR8	*U. uncialis*	sporulated
CORR10	*U. uncialis*	sporulated
CO7	*U. uncialis*	sporulated
CAL10	*U. uncialis*	sporulated
BLD17	*U. australis*	16.1±3.1^a^
HALAUS2	*U. australis*	15.5±1.0^a^
SIL32	*U. compressa*	15.2±0.5^a^
TRAM84	*U. fenestrata*	sporulated
CAL7	*U. rigida*	sporulated

##### Species identification and selection process

Although the CAPS assay is not fully quantitative because it relies on the amplification of the ITS1, which is part of the 45S rDNA repeats whose number can vary from one species to another (Rogers and Bendich 1987), it can nevertheless be considered semi-quantitative and allows us to identify the presence of different species in bulk biomass down to ~6.5% by tissue weight (Fort et al. 2021). The results of species detection over the 70 days of cultivation are presented in Table 3.

**Table 3:** Species identification of *Ulva* bulk biomass at 15 ppt and 35 ppt over the 70 days of the garden experiments. Raw data (gel pictures) are provided in Supplementary Fig. S1

Days	0		32		36		39		49		56		63		70	
Salinity (ppt)	15	35	15	35	15	35	15	35	15	35	15	35	15	35	15	35
*U. australis*	+	+	+	+	+	+	+	+	+	+	+	+	+	+	+	+
*U. compressa*	+	+	+													
*U. fesnestrata*	+	+														
*U. lacinulata*	+	+														
*U. rigida*	+	+														
*U. uncialis*	+	+														

At 35 ppt, *U. australis* was the only species detected after 32 days of culture. As all species were represented by strains that survived when cultivated individually at 35 ppt (Table 1), the relative increase in *U. australis* biomass is thought to be mostly driven by its higher SGR. This can be seen when predicting the relative abundances in mixed culture from the growth rates determined from individual cultures (Supplementary Table S2 - A - 35 ppt). *Ulva australis* was predicted to represent ~64% of the biomass at day 32, with *U.* *compressa* only ~14%, due to a lower number of strains at start of the experiment and despite similar SGRs. These predictions were based on mean growth rates and if we consider the standard deviations, *U.* *compressa* percentage could fall below the CAPS assay detection threshold (Supplementary Table S2 - B – 35 ppt - maxSGR for BLD17).

At 15 ppt, both *U. australis* and *U.* *compressa* *were* detected at 32 days. The failure to detect other species (*U.* *uncialis*, *U.* *lacinulata*, *U.* *fenestrata*, and *U. rigida*) was unsurprising given their lack of tolerance to low salinity in individual culture. Hence, the strain selection at 15 ppt was mostly based on survival. *Ulva compressa* was not detected by the CAPS assay at day 36 although based on individual growth data, it should still have represented around 18% of the total biomass (Supplementary Table S2- C - 15 ppt). When considering the standard deviations, it can fall to 9% of the total biomass, which bring it close to the threshold of CAPS assay detection (Supplementary Table S2- D - 15 ppt - maxSGR for BLD17).

Our findings are largely consistent with growth rate and survival driven competition, both at 35 ppt and 15 ppt, and are a strong indication that mixed cultivation is an effective strategy to isolate fast-growing strains suited to diverse environments.

### Biochemical composition

**Fig. 2:**
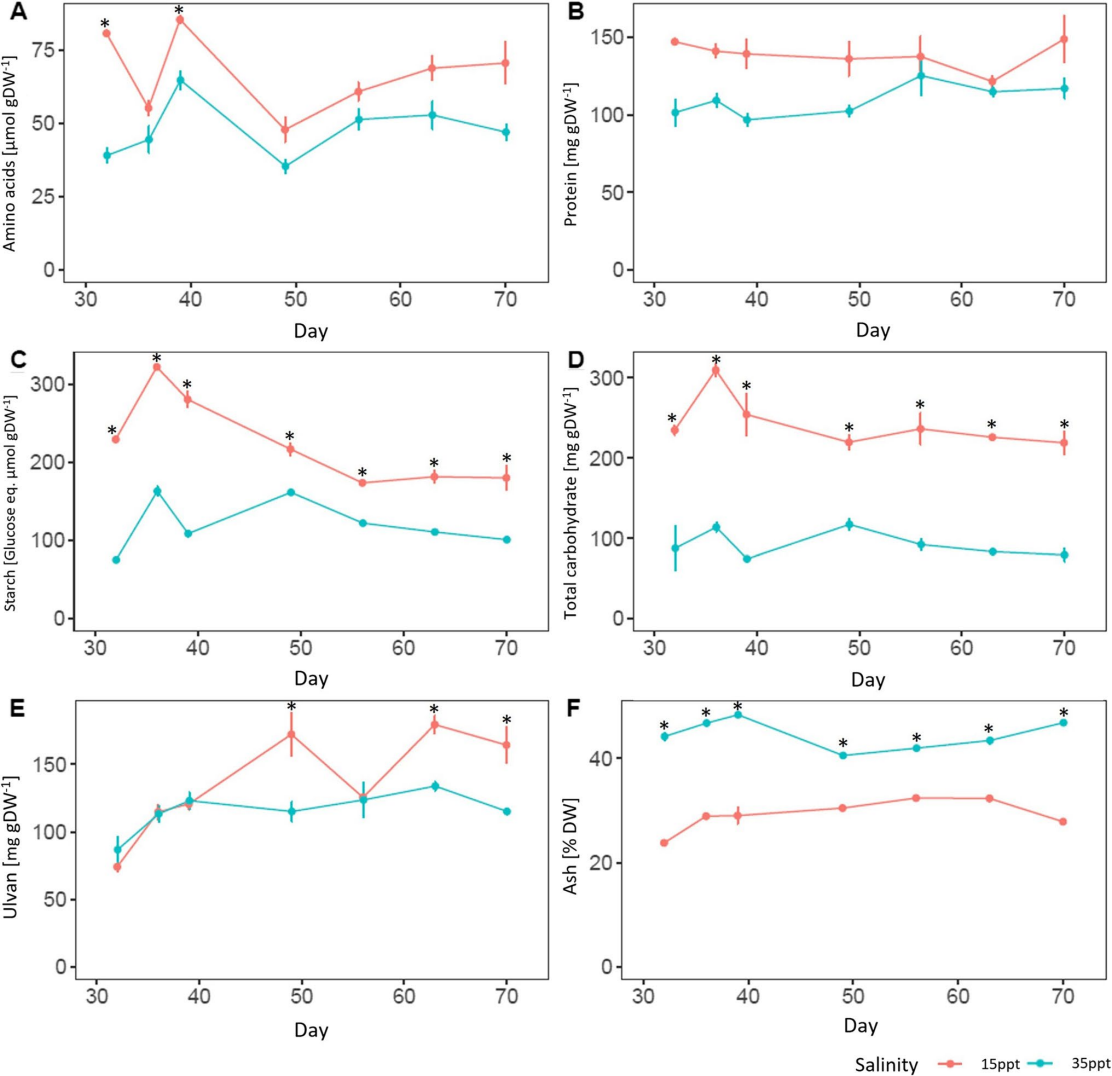
Biochemical content of the bulk biomass from 32 days of culture to 70 days at both salinities; seawater (35 ppt) and brackish water (15 ppt); **(A)** Amino acids content (μmol gDW^-1^±SD); **(B)** Protein content (mg gDW^-1^±SD); **(C)** Starch (Glucose eq. μmol gDW^-1^±SD); **(D)** Total carbohydrate content (mg gDW^-1^±SD); **(E)** Ulvan content (mg gDW^-1^±SD); **(F)** Mineral (ash) content (% DW ± SD), Data represents mean in proportion of dry weight (DW) ±SD, n=3. Asterisks indicate significant difference between salinities (Two-way ANOVA, *p* <0.05)

Biochemical analyses were performed after 32 days of cultivation, so when the number of species present was reduced and *U. australis* was the dominant species at both salinities (Table 1). Significant differences in biochemical composition were observed between the two salinities used. The content of total carbohydrates was significantly higher at 15 ppt than at 35 ppt at all time points, about twice more at 15 ppt. Total carbohydrates, determined in the insoluble fraction after ethanolic fraction, are mostly composed of ulvans, starch and insoluble fibers. In agreement, we also saw an increase in starch (all time points) and ulvans, (three time points) at 15 ppt compared to 35 ppt (Fig. 2). Free amino acids and pigments also showed a tendency for an increase in content at 15 ppt, with some timepoints being significant (Supplementary Figure S2). No significant difference in protein content was found between the two salinity treatments

The general increase in the biochemical compounds we determined, which represent a large part of the total organic matter present in the biomass, could be explained by (1) a general over-accumulation of metabolites at low salinity due to active physiological processes or (2) a decrease in the mineral content (ash), which led to a proportional increase of the fraction of organic biomass. The ash content was significantly lower in the low salinity biomass, with an average of 29% DW, compared to 45% DW for the biomass grown at 35 ppt (Fig. 2F).

**Fig. 3:**
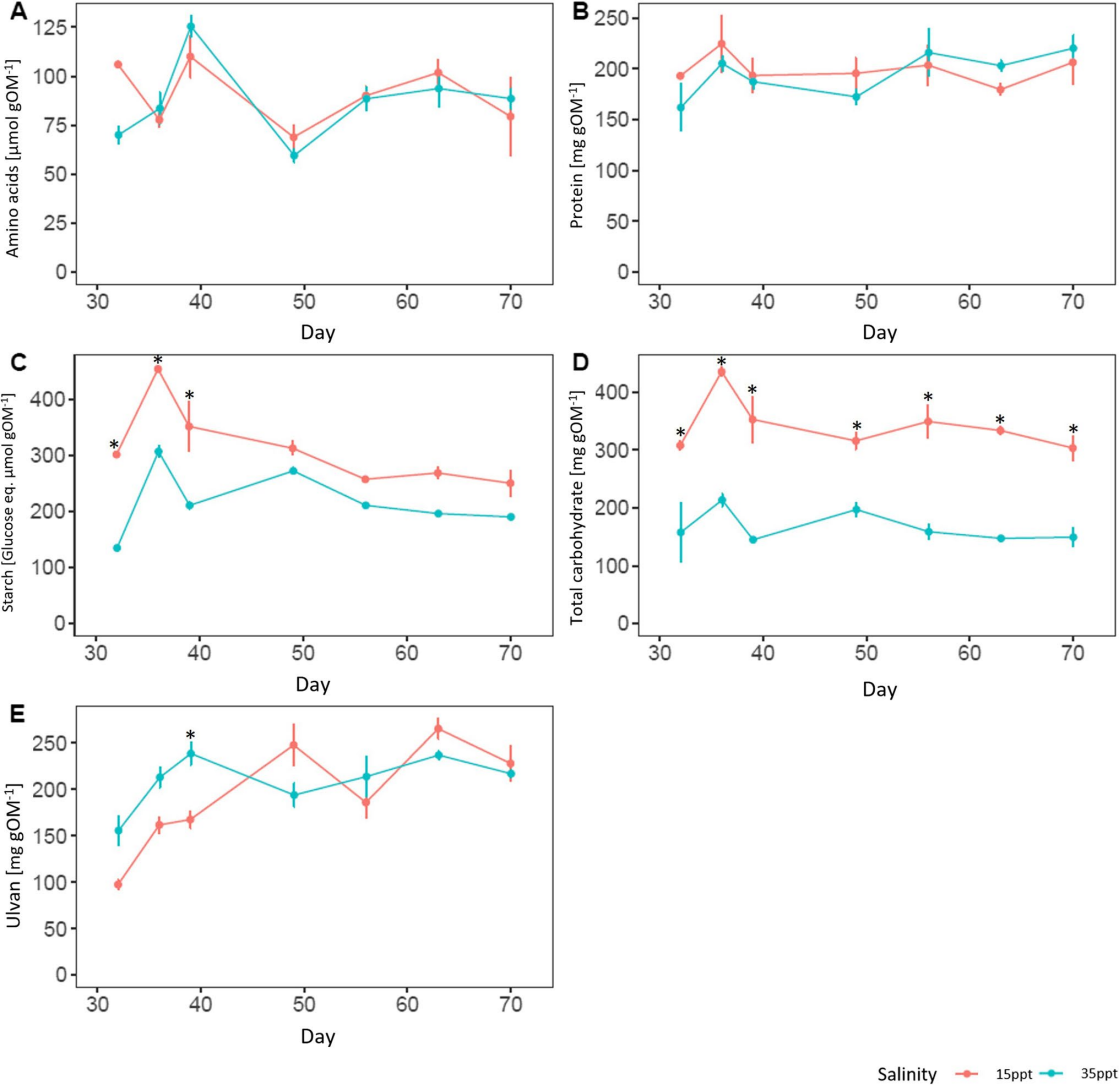
Metabolite content expressed on organic matter basis of *Ulva* biomass grown in seawater (35 ppt) and brackish water (15 ppt); **(A)** Amino acids content (μmol gOM^-1^±SD); **(B)** Protein content (mg gOM^-1^±SD); **(C)** Starch (Glucose eq. μmol gOM^-1^±SD); **(D)** Total carbohydrate content (mg gOM^-1^±SD); **(E)** Ulvan content (mg gOM^-1^±SD). Data represent means in proportion of organic matter (OM) ±SD, n=3. Asterisks indicate significant difference between salinities for each timepoint (Two-way ANOVA, *p* <0.05)

To test if low salinity induced specific changes in some biochemical compounds, we expressed the contents in organic compounds on an organic matter basis (Fig. 3). No significant differences were observed for the proteins and amino acids contents (*p*>0.05). However, the content of carbohydrates and starch (only for the 32-, 36- and 40-day timepoints) were significantly higher at low salinity compared to high salinity (Two-way ANOVA, *p*-value<0.05), suggesting that low salinity induced specific changes in *Ulva* metabolism, particularly carbohydrates. The common-garden experiment results showed that after one month of culture, the individuals maintained the same growth rate (Fig. 1A). These results, together with the biochemical results, show that at low salinity, the species *U.* *australis*, for the same growth rate, tends to accumulate more organic matter. In other words, it means that growth expressed as an accumulation of organic matter, was higher at 15 ppt than at 35 ppt.

## Discussion

### Common-garden experiment allows rapid strain selection process through competition and survival

The objective of this study was to develop a method to select the best performing and ideally fastest growing strains under specific conditions. The fastest-growing strain is predicted to dominate in mixed cultures according to the principle of competitive exclusion, which states that in the absence of stabilizing interactions, a community of species competing for the same ecological niche will be overtaken by its fastest-growing member (Hardin 1960).

The effects of intraspecific competition on algal biomass and the development of natural populations have already been described (Schiel and Choat 1980; Schiel 1985; Reed 2010) and in particular for *Ulva* species known to be responsible for the “green tide phenomenon”, a seasonal phenomenon involving a large volume of the marine environment being invaded by *Ulva* species, due to anthropogenic causes, which is considered to be a highly competitive environment (Fort et al. 2020). However, the process of selection/competition under artificial conditions is less documented (Creed et al. 1998). If competition becomes sufficiently severe, the death of suppressed individuals occurs (self-thinning), which can be seen as the ultimate manifestation of intraspecific competition (Steen 2004; Nabivailo and Titlyanov 2006). It is always assumed that slower growing individuals are more likely to die in macroalgae populations and that the mechanism responsible is “dominance and suppression”. Nabivailo and Titlyanov (2006) described that this process of competition between artificial algal communities can occur through different mechanisms:

1. the exploitation of the environment, i.e., the more intensive uptake and efficient use of limited resources by one species, which prohibits the use of these resources by other individuals,
2. the occupation of space by the rapid overgrowth of one species,
3. chemical influence, i.e., the allelopathic inhibition of the growth and photosynthesis of associated algal species by production of secondary metabolites such as phenolic and halogenated compounds. However, although some studies have shown that *Ulva* species (*U. australis* and *U.* *linza*) can exhibit negative allelopathic effects on microalgal communities, such allelopathic effects have never been described within *Ulva* species (Nan et al. 2004, 2008; Jin et al. 2005).

In this study, to validate this selection method, multiple strains, for which individual growth rates were predetermined, were co-cultivated with biomass periodically removed after a density of culture >3g L^-1^ was achieved. Two conditions were evaluated, i.e., saline water (35 ppt) and brackish water (15 ppt), under a light intensity of 200 μmol photons m^-2^s^-1^, 12h:12h photoperiod, 15 °C and nutrient rich medium (f/2 medium). The results show that, after 32 days of co-cultivation, most of the species could no longer be detected in either cultures. This was before any biomass removal from the culture.

After 36 days, regardless the salinity, *U. australis* was the only species detected. These two *U. australis* strains, which exhibit the highest growth rate when grown individually, are the only ones to come from a "green tide" environment. This "green tide" condition has been described as a competitive environment that selects for fast-growing *Ulva* strains, which could explain these results (Fort et al. 2020). Selection through competition based on growth performance is therefore demonstrated in these experiments, as well as strain adaptation to low salinity. According to our results, growth rate was revealed to drive the selection but survival also appears important, especially at low salinity. Under this condition, neither survival nor growth could clearly explain our results. Hence, competition might have occurred through other means, like direct interactions between strains (i.e., occupation of space, and/or allelochemical influence). The selection is also likely to be influenced by less predictable effects on survival and growth, such as developmental phases. Even if these individuals were maintained in vegetative growth, a difference in developmental age cannot be ignored and must be considered as a factor impacting selection (Park 2020). However, these events are likely to be synchronized within species, like the observed stimulation of reproduction within the species *U.* *lacinulata* during this present experiment.

It is also important to mention that our common-garden experiment was not replicated for each treatment. We cannot exclude that replicating the experiment might have led to slightly different results. However, considering that the strains which were selected during the experiment were among those which showed the highest growth rates when grown individually, we expect that a repeat of these garden experiments will lead to similar results.

Above all, this batch selection method represents a significant advance in improving the technology of the aquaculture industry and can be implemented on a larger scale. Indeed, even though this study was conducted in the laboratory under controlled conditions with a limited number of replicates and individuals, the extension to a larger scale and to outdoor culture can be achieved relatively easily. Even if batch and continuous culture is already well developed in macroalgae aquaculture, the use of free-living batch culture for a purpose of strains selection have never been properly described in macroalgae studies (Henley 2019). This selection process is already common in single-celled microorganisms like bacteria and yeast (Herring et al. 2006; Barrick et al. 2009; Selmecki et al. 2015; Woronoff et al. 2020; Ratib et al. 2021) and is often associated with the selection of mutants outperforming other cells. Here, the use of common-garden experiment to select existing genotypes with a potential advantage for commercial exploitation has proved to be of minimal effort.

### Long- term low salinity impact on *Ulva* growth

The individual strain experiment allowed us to characterise the impact of the salinity on the growth of *Ulva* spp. (Table 2). These results showed that the low salinity conditions induced rapidly the sporulation and ultimately death of the majority of *Ulva* species tested in this study (*U.* *fenestrata*, *U.* *lacinulata*, *U.* *uncialis*) died within one month of low salinity treatment). *Ulva* *australis* and *U.* *compressa* were the only species performing well at low salinity for more than two months. In large agreement, only *U. australis* was able to perform well for more than two months at low salinity in the common garden experiment. This allowed us to study the impact of reduced salt concentration on the growth and nutritional properties of the species *U.* *australis*. It has already been shown that *U. compressa* with a foliose thallus can grow in a hyposaline environment in contrast to *U. australis* where the results differ between studies. Indeed, Floreto et al. (1993) demonstrated that *U. australis* is unable to develop and maintain a proper metabolism in a hyposaline environment (at 20 ppt). In contrast, Choi et al. (2010) have shown that under non-limiting nutrient and light conditions, growth rates are highest in the 15-25 ppt salinity range, with an optimum between 15 and 20 ppt. Those differing results might be explained by intra-specific variation within *U. australis* (Fort et al. 2019). Concerning the species *U. compressa*, some ecological surveys have already described this species as having two distinct morphotypes, tubular and foliose thalli, these can be found in low salinity-brakish environment such as estuaries and brackish lakes (Ogawa et al. 2013; Steinhagen et al. 2019; van der Loos et al. 2022). *Ulva compressa* with foliose morphotype was first discovered in brackish water in the Ythan Estuary in Scotland (Tan et al. 1999). However, Wu and Lee (2008) demonstrated that the growth of *U.* *compressa* is affected by extreme low salinity and the highest growth rates was found around 20 ppt. Considering these different observations, it appears therefore difficult to affirm that one species is more tolerant to variations in salinity than another. Indeed, the genetic background of each individual has an important impact on its survival and the ability to adapt to any environmental condition. Intra-species variation in *Ulva* species is extremely large, and as a result we can not really make conclusions at a species level (Lawton et al. 2013; Fort et al. 2020). This study demonstrates that not all marine *Ulva* foliose strains are equal in their ability to adapt and thrive under hyposalinity. Strains from two species, *U. compressa* and *U.* *australis*, were able to modify and regulate their metabolism to adapt to long-term brackish environment, which is a very promising result for the use of *Ulva* species in environments where low salinity is present such as estuaries or for the treatment of municipal/agricultural wastewaters.

### Increase in metabolite content in *Ulva* under low salinity treatment

After the characterization of the growth, we then investigated the impact of low salinity on the biochemical composition of mostly *U.* *australis* as the common-garden experiment selected for this species before collecting the biomass (32 days) or a few days later (36 days). Low salinity resulted in significant accumulation of organic compounds, (total carbohydrate, starch, ulvan and pigments).

Few studies have reported an accumulation of metabolites or compounds in *Ulva* species when salinity changes (Kakinuma et al. 2006; Fort et al. 2024). Kakinuma et al. (2006) observed that *U.* *australis*, when exposed to low salinity was accumulating photosynthetic pigments from the first day of treatment. However, the authors also observed a significant decrease in growth for up to 5 days upon exposure and attributed those observations to a disturbance of the carbon/nitrogen metabolism. In the present study, after two months of cultivation the growth of *U.* *australis* was no longer negatively affected by low salinity, so we can therefore consider that the strains were fully adapted. It has also been shown in freshwater microalgae (*Chlorella* spp., *Scenedesmus* spp., *Arthrospira* spp.) that under salt stress, the microalgal cells adapt by regulating their internal osmotic pressure through the accumulation of organic metabolites (so-called compatible solutes) and the modification of physiological and biochemical processes (Ben-Amotz and Avron 1983; Warr et al. 1985; Kirrolia et al. 2011; Bajwa and Bishnoi 2015). But in this case, it is about increasing cell osmotic pressure in order to respond to an increase in salt concentration in the external media, so the opposite of our treatment. Indeed, we found that the total organic matter per gram of biomass increases when the salinity decreases and thus we are not observing an increase in organic matter due to the requirement to increase osmotic pressure within cells. Accordingly, the main organic compounds accumulating in response to low salinity were starch and ulvans, which do not have an osmotic role. However, a recent study demonstrated that a short-term (seven days) low-salinity treatment could have a significant impact on the lipid profile of certain *Ulva* species (Fort et al. 2024). It has been observed that this low-salinity treatment (7.5 ppt and 15 ppt) led to an increase in the fatty acid (polyunsaturated and ω-3 fatty acids) and lipid (galactolipids, betaine lipids and certain phospholipids) content of *U. australis* species. In this study, we identified the same accumulation response to low salinity for the carbohydrates content. Moreover, amino acid and protein contents were neither increased nor decreased by low salinity treatment showing that the increase in carbohydrate content was not the result of a decrease in the other organic metabolites. This result could prove advantageous for *Ulva* biomass valorisation. Indeed, the treatment of *Ulva* at low salinity would make it possible to obtain a more valuable biomass with higher concentrations of organic matter and in particular carbohydrates without compromising other biochemical compounds such as proteins. Such process could become important to increase the marketability of seaweeds as healthy nutritional product, but also for biofuels and bioplastics which are made of carbohydrates. In addition, this low salinity treatment is interesting because lower mineral content and higher metabolite content per unit of biomass can improve and facilitate different processes/extractions such as those in use for the pharmaceutical and biofuel industries. On a long term, an understanding of the mechanisms of salinity tolerance in *Ulva* species and its effect on chemical composition would facilitate strain selection and future breeding programmes. Such selection would allow to develop the use of *Ulva* as a biofilter for terrestrial wastewater bioremediation which require *Ulva* strains able to withstand low salinity environments for long periods.

## Conclusion

In this study we demonstrated that common-garden experiments are suited for the selection of strains adapted to given environmental conditions. During this study, the selection was complete after 5 weeks of cultivation. The time needed for selection will however depend on the specific parameters tested, i.e., the growth conditions and the strains themselves. This batch free-living culture technique therefore allows the aquisition, without much effort and time in comparison to individual phenotyping, of an individual that is tailored to the desired conditions and applications. This method has been tested under low salinity and allowed us to show the ability of *U. australis* strain(s) to grow and adapt their metabolism at low salinity over the long-term. A limitation of our method is that we were not able to identify within our culture the different strains of a given species. This limitation could be overcome in future via the development of molecular markers, e.g., single nucleotide polymorphisms or microsatellites. When available, common-garden experiments could be used to study in detail the mechanisms involved in the competition process. The common-garden experiment is a new, fast and inexpensive method of selecting the fastest individual under certain conditions. Importantly, this method is accessible to any lab/aquaculture industry.

### Supplementary Information

Below is the link to the electronic supplementary material.

Supplementary file1 (XLSX 17 KB)

Supplementary file2 (DOCX 2346 KB)

Supplementary file3 (XLSX 10 KB)

Supplementary file4 (XLSX 61 KB)

## Data Availability

Materials will be made available on request and data are available in Supplemental data.
