# Supplementary material for: Fast screening method to identify salinity tolerant strains of foliose Ulva species. Low salinity leads to increased organic matter of the biomass
Source: J Appl Phycol. 2024 Apr 3;36(4):2161–72. doi: 10.1007/s10811-024-03222-0 (PMC11263424; doi:10.1007/s10811-024-03222-0)
Supplement: Supplementary file 2 — Supplementary file2 (DOCX 2346 KB) [file 10811_2024_3222_MOESM2_ESM.docx]

*Figure S2. Gel pictures of the caps assay for bulk species determination from the Ulva garden experiment*

1- BtscI +T7

2- CvIQI+BtscI

3- CvIQI+BtscI+T7

4-  BfaI+ BamHi

5- BfaI+ BamHi +T7

6- PspomI

7- PspomI +T7

1.
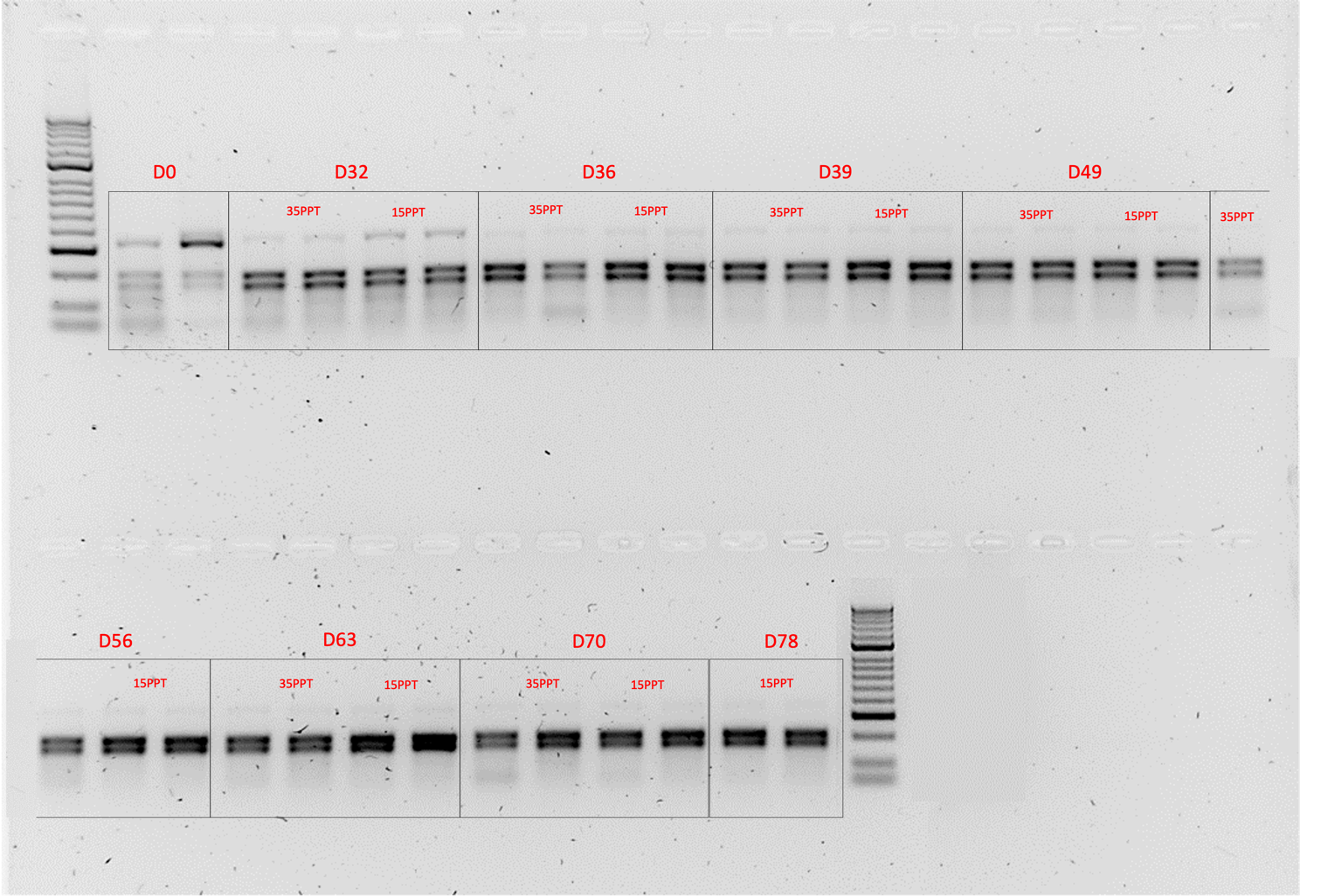
Gel picture of the ITS1 digestion with enzyme BtscI and the endonuclease T7
2. Gel picture of the ITS1 digestion with enzymes CviQI and BtscI


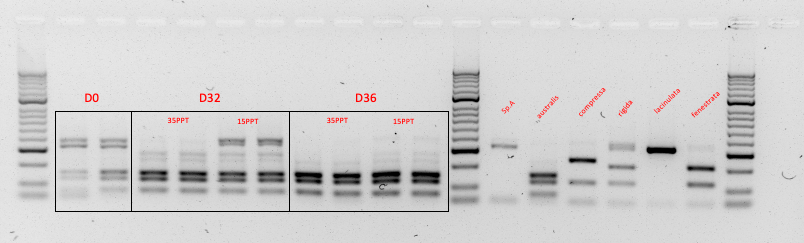


1. Gel picture of the ITS1 digestion with enzyme CvIQI, BtscI and endonuclease T7


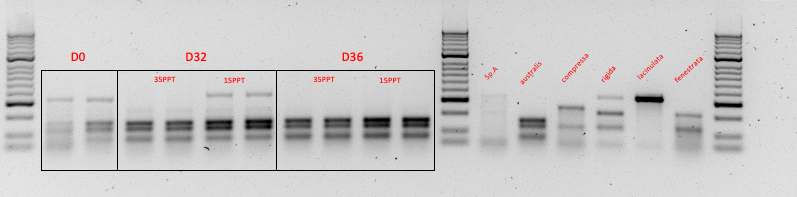


1. Gel picture of the ITS1 digestion with enzymes BfaI and BamHI


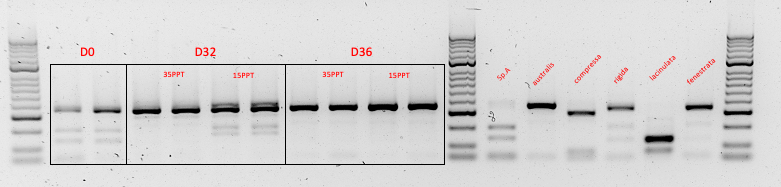


1. Gel picture of the ITS1 digestion with enzyme BfaI, BamHi and the endonuclaseT7


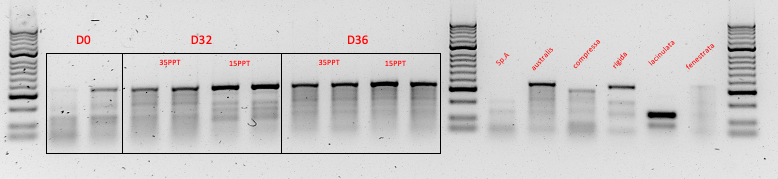


1.
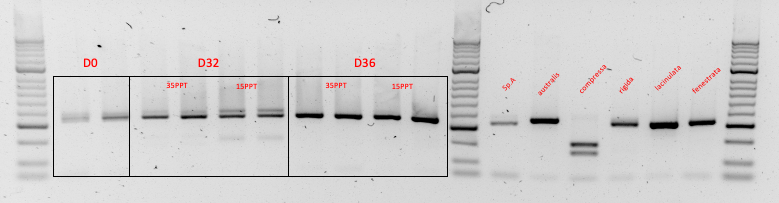
Gel picture of the ITS1 digestion with enzyme PspOMI
2.
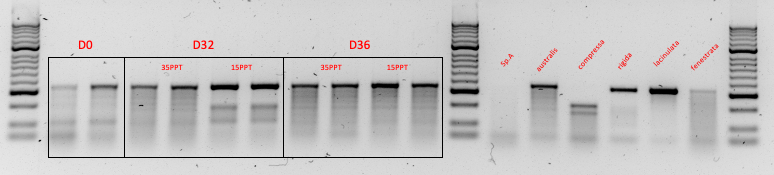
Gel picture of the ITS1 digestion with enzyme PspOMI and endonuclease T7

*
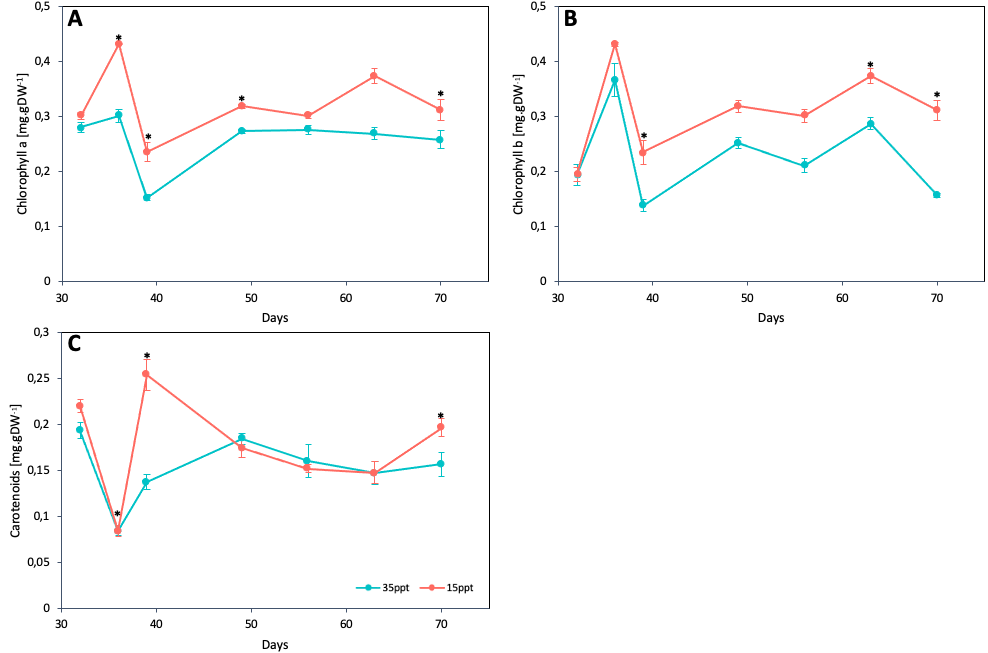
Figure S2. Pigment content of the batch* Ulva *culture during the 70 days of cultivation at two salinities; seawater (35 ppt) and brackish water (15ppt);* ***A****: Chlorophyll a content (mg.gDW^-1^±SD); ;* ***B****: Chlorophyll b content (mg.gDW^-1^±SD);* ***C****: Carotenoid content (mg.gDW^-1^±SD). Data represents mean in proportion of dry weight ±SD, n=3. Asterisks indicate significant difference between salinities (Two-way ANOVA, p-value<0.05).*
